# Chronic Intermittent Hypoxia Exposure Induces a Unique Microglial Transcriptome in 5XFAD Mice

**DOI:** 10.1007/s12035-025-05390-7

**Published:** 2025-11-19

**Authors:** Kaitlyn M. Marino, Andrea C. Ewald, Jaidynne N. Lash, Erin C. McCann, Shibani Ram, Phinea Z. Romero, Tao Wang, Abigail L. Watters, Abigail B. Radcliff, Daniel C. Shippy, Tracy L. Baker, Tyler K. Ulland, Jyoti J. Watters

**Affiliations:** 1 Neuroscience Training Program, University of Wisconsin-Madison, Madison, WI USA; 2 Department of Pathology and Laboratory Medicine, University of Wisconsin-Madison, Madison, WI USA; 3 Department of Comparative Biosciences, University of Wisconsin-Madison, Madison, WI USA; 4 Wisconsin Alzheimer’s Disease Research Center, Madison, WI USA

**Keywords:** Neuroinflammation, Intermittent hypoxia, Microglia, Amyloid

## Abstract

Clinical observations suggest that obstructive sleep apnea (OSA) and Alzheimer’s disease (AD) pathology may be linked; however, causal mechanisms and relationships are unclear. To investigate the potential interaction between amyloidosis and intermittent hypoxia (IH), a hallmark of OSA, starting at 4 months of age 5XFAD mice were exposed to chronic IH (CIH) consisting of 20 episodes per hour of hypoxia for 12 h/day, daily for 4 months (males) or 6 months (females). CIH did not induce significant changes in amyloid burden or the number of astrocytes in males or females, but there was a slight decrease in the number of microglia observed in the cortex of 5XFAD mice of both sexes. To further explore this effect, we performed bulk RNA sequencing on isolated microglia. In WT mice, the most robust gene changes induced by CIH were identified in male microglia, many of which were pro-inflammatory. In microglia from 5XFAD mice, compared to normoxia (NX), CIH exposure induced comparatively more DEGs in males. Further, in genes that were upregulated by CIH in WT vs 5XFAD mice of both sexes, there was an enrichment of pathways associated with oxidative phosphorylation, aerobic and cellular respiration, and ATP synthesis. These changes indicate that CIH has a more robust effect on the microglial transcriptome in 5XFAD mice than in WT mice, suggesting that the synergy between AD and OSA pathologies may be driven by metabolic changes in the microglial transcriptome. These observations are particularly interesting given the known sex differences in OSA and AD pathology in human disease.

**Supplementary Information:**

The online version contains supplementary material available at 10.1007/s12035-025-05390-7.

## Introduction

As the world’s population ages, identifying and leveraging known risk factors associated with cognitive decline are of increasing importance as they could lessen the significant financial, medical, and caregiver strains on society. Targeting chronic diseases that can become a co-morbidity to diseases of aging, like Alzheimer’s disease (AD) and related dementias, later in life may acutely improve patient health and modulate disease severity or onset. One of these potential co-morbidities is obstructive sleep apnea (OSA), which affects an estimated 1 billion people worldwide [1]. OSA is a form of sleep disordered breathing in which the airway is partially or completely blocked multiple times per hour during sleep, resulting in periods of intermittent hypoxia, hypercapnia, and sleep fragmentation [2]. Untreated, OSA can result in daytime sleepiness, fatigue, and diminished cognition [3, 4]. The primary treatment for OSA is the use of a continuous positive airway pressure (CPAP) device, worn during sleep, that keeps the airway open to help regulate blood gases. CPAP is effective in reducing symptoms and improving perceived health-related quality of life, but there is no evidence of significantly improved cognition in those with mild to moderate OSA [5–9]. CPAP is only effective, however, if individuals are willing to use it, but only an estimated 30–60% of patients reliably use their CPAP devices, despite recent advances that make the equipment more comfortable and user-friendly [10–12]. Neuroimaging studies have been used to probe the impact of OSA symptomology on brain structure. OSA correlates with thickening of several grey matter areas and can be stratified by the primary symptom type (hypoxia vs sleep disturbance) [13–15]. Also, white matter changes and reduced attention are exacerbated in individuals over 60 years old with severe OSA [16, 17]. Correlations between OSA and dementia have been identified [18, 19], so there is increasing interest in OSA as a potential contributor to neurodegenerative disorders, including AD [20, 21].

AD is a progressive disease thought to be driven by amyloid β (Aβ) plaque deposition, beginning decades prior to symptom onset, and the subsequent formation of neurofibrillary tau tangles (NFTs) [22]. A recent meta-analysis found that OSA was associated with elevated AD biomarkers in both the blood (total amyloid, Aβ_40_, Aβ_42_, and total tau) and cerebrospinal fluid (CSF) [23]. Recent lipidomic analyses have identified a unique signature in the CSF and blood of individuals with AD and severe comorbid OSA [24, 25] that is thought to be mediated by hypoxia-mediated inflammation, although additional studies are needed to confirm this. Further, positron emission tomography (PET) neuroimaging shows increased levels of amyloid in individuals with OSA [26]. APOE ε4, a major genetic risk factor for AD, is a secondary risk factor for cognitive decline, which is exacerbated with worsening OSA severity [27, 28]. Both OSA and AD cause neuroinflammation; while the mechanism for each pathology alone is thought to involve the activation of Toll-like receptor-4 (TLR4), the NACHT-, LRR-, and pyrin (PYD)-domain-containing protein 3 (NLRP3) inflammasome, ASC speck formation, and astrocyte activation, the cellular interaction between these pathologies remains unclear [29–33].

Given the paucity of sufficiently powered human studies, and the large overlapping populations between AD and OSA, we and others have turned to mouse models to examine the intersection of these disorders. One study examined the synergism of these pathologies using 1 month of CIH exposure, and found increased markers of astrocyte activation in the APP/PS1 mouse model, another AD-like cerebral amyloidosis mouse model [34]. Here, we employed the 5XFAD mouse model of AD [35] which develops rapid accumulation of amyloid pathology, and we exposed males and females to CIH for 4 or 6 months, respectively, to investigate the long-term effects of CIH on the glial response to amyloid pathology and disease burden.

## Materials and Methods

### Animal Care and Use

Wildtype (WT) females (C57BL/6J; JAX Stock #000664) were crossed with 5XFAD [35] male mice obtained from the Mutant Mouse Resource and Research Center at JAX (B6SJL-Tg (APPSwFlLon, PSEN1*M146L*L286V)6799Vas/Mmjax; JAX Stock #034840) and group housed in the University of Wisconsin-Madison School of Veterinary Medicine vivarium according to UW-Madison IACUC approved protocols #V005173 and #M006091. Mice were maintained on a 12-h light/12-h dark cycle with food and water provided ad libitum. Animals were anesthetized and perfused with cold Phosphate Buffered Saline (PBS) before tissue preparation. Females were harvested at 10 months of age while males were harvested at 8 months of age due to increasingly aggressive behavior that prevented group housing beyond this time.

### Chronic intermittent hypoxia (CIH)

Beginning at 4 months of age and until they reached the study endpoint, a total of 4 months and 6 months of treatment for males and females, respectively, were administered. Mice were exposed to intermittent hypoxia (IH) or room air (normoxia, NX) during their 12-h period of presumptive sleep, as described previously [33]. IH was achieved by alternating inspired oxygen levels between 6% O_2_ and 21% O_2_ over 90-s intervals. Mice subjected to NX conditions were exposed to the same noise and airflow as IH-exposed mice, but inspired O_2_ levels were consistent at 21% O_2_.

### Microglia Isolation

As described previously [36, 37], following cardiac perfusion, cerebral brain tissue was mechanically dissociated and placed in cold HBSS (Fisher #12–022-CM). Samples were then enzymatically digested with papain (from papaya latex, Sigma #P4762-500MG) and DNAse (Invitrogen #12185010) at 37 °C, gravity fed through filters and myelin was separated from the cells using 26% percoll solution (Cytiva #17089101) and centrifuged at 2100 rpm, at 4 °C for 15 min. The pellet was resuspended and incubated with anti-CD11b magnetic beads (Miltenyi Biotech #130-093-634) in IMAG (0.5% BSA and 2 mM EDTA in PBS) and run through primed magnetic columns (Miltenyi Biotech catalog #130-042-201). The isolated microglia were stored in TriZol (Invitrogen, 15596026) at −80°C.

### RNA Isolation and Sequencing Analyses

RNA isolation from n = 5 independent samples for each sex, genotype, and treatment was performed per the manufacturer’s instructions (Invitrogen, 15596026). Total RNA was sent to Novogene USA for bulk mRNA sequencing (NovaSeq6000 PE150, Illumina) and was aligned to the GRCm38/mm10 mouse genome. As we have reported previously [38], gene count files were imported into R and genes with a CPM > 1 in 3 samples were included. Differential expression analysis was calculated between groups using EdgeR [39]. PCA plots were used to identify outliers (Fig S1-2) One female normoxia sample was identified as an outlier in several comparisons. Statistically significant comparisons had a false discovery rate (FDR) < 5% and a log_2_FC > 1. Gene ontology analyses were completed using the PANTHER Overrepresentation test [40] within the STRING analysis tool [41] using a high confidence (minimum required interaction score = 0.7); like GO terms are grouped at a similarity of 0.8.

### Tissue Fixation and Immunofluorescence Staining

Following perfusion with cold PBS, tissue was fixed in 4% paraformaldehyde for 48 h, rinsed with PBS, and sunk in 30% sucrose for 48 h. Tissue was oriented and then frozen in a 2:1 30% sucrose/Tissue Tek OCT compound (Sakura #4583) solution and stored at −80°C. Brains were sectioned into 40 µm (Leica) sections and stored in cryoprotection buffer (30% ethylene glycol, 30% sucrose in PBS). Sections were selected, blocked in confocal blocking buffer (3% BSA, 0.15% Triton-X100 in PBS) for 1 h and incubated with primary antibody to Iba-1 (Abcam ab5076; 1:1750) or GFAP (Alexa Fluor 488 Conjugated, Invitrogen 53–9892-82; 1:1000) overnight at 4°C. Floating sections were rinsed with PBS and incubated at room temperature with Alexa Fluor 488 Donkey anti-Goat secondary antibody (Abcam ab150129; 1:500), Methoxy-X04 (Tocris Biological #4920; 1:1000), and TO-PRO-3 (Thermo Fisher #T3605; 1:1000) in confocal blocking buffer. Sections were rinsed with PBS and mounted with Fluoromount G (SouthernBiotech, Cat. No. 0100-001) and #1 coverslips (Fisherbrand, Cat. No. 12541035) on superfrost slides (Fisherbrand Cat. No. 12–550-15).

### Confocal Microscopy

Immunofluorescent stained slides were imaged on a Nikon A1R+ in the Optical Imaging Core at the University of Wisconsin-Madison. Images were obtained as Z-stack using a 20X objective (NA = 0.75) at a step depth of 0.7 µm for each 40 µm section (at least 3 sections per animal).

### Imaris Analysis

As described previously [42], confocal images were loaded into Imaris (Bitplane 9.5.1) for analysis. The Iba-1 or GFAP (microglia or astrocyte respectively) and TO-PRO-3 channels were colocalized, and used to obtain a cell count and spatial coordinates for cells in the 3-dimensional images using the “spots” function. Plaque analyses were completed by generating 3-dimensional surfaces of methoxy-X04^+^ plaques. The number of spots within 30 µm of all plaque surfaces in the image was then calculated.

### Plaque Quantification in the Cortex

Tiled, single-plane images of methoxy-X04 staining were obtained on a Zeiss Imager.M2 microscope and exported into ImageJ [43] as a TIFF file. Cortex ROIs were traced for each section. A percentile threshold (0.4% (females) or 0.5% (males)) and a particulate size threshold of 100 pixels were used to quantify the percent area occupied by plaques.

### Statistics

Males and females were analyzed separately for all experiments. Statistical analyses were completed in Graph Pad Prism (Dotmatics) with a significance threshold of *p* < 0.05 for two-tailed unpaired t-tests and 2-way ANOVAs. Tukey post-hoc comparison tests were used for multiple comparisons at the same significance threshold. ROUT testing was used to identify and remove outliers (Q = 1%).

## Results

### CIH Decreases Body Mass in Male 5XFAD Mice but Increases It in Female 5XFAD Mice

**Fig. 1:**
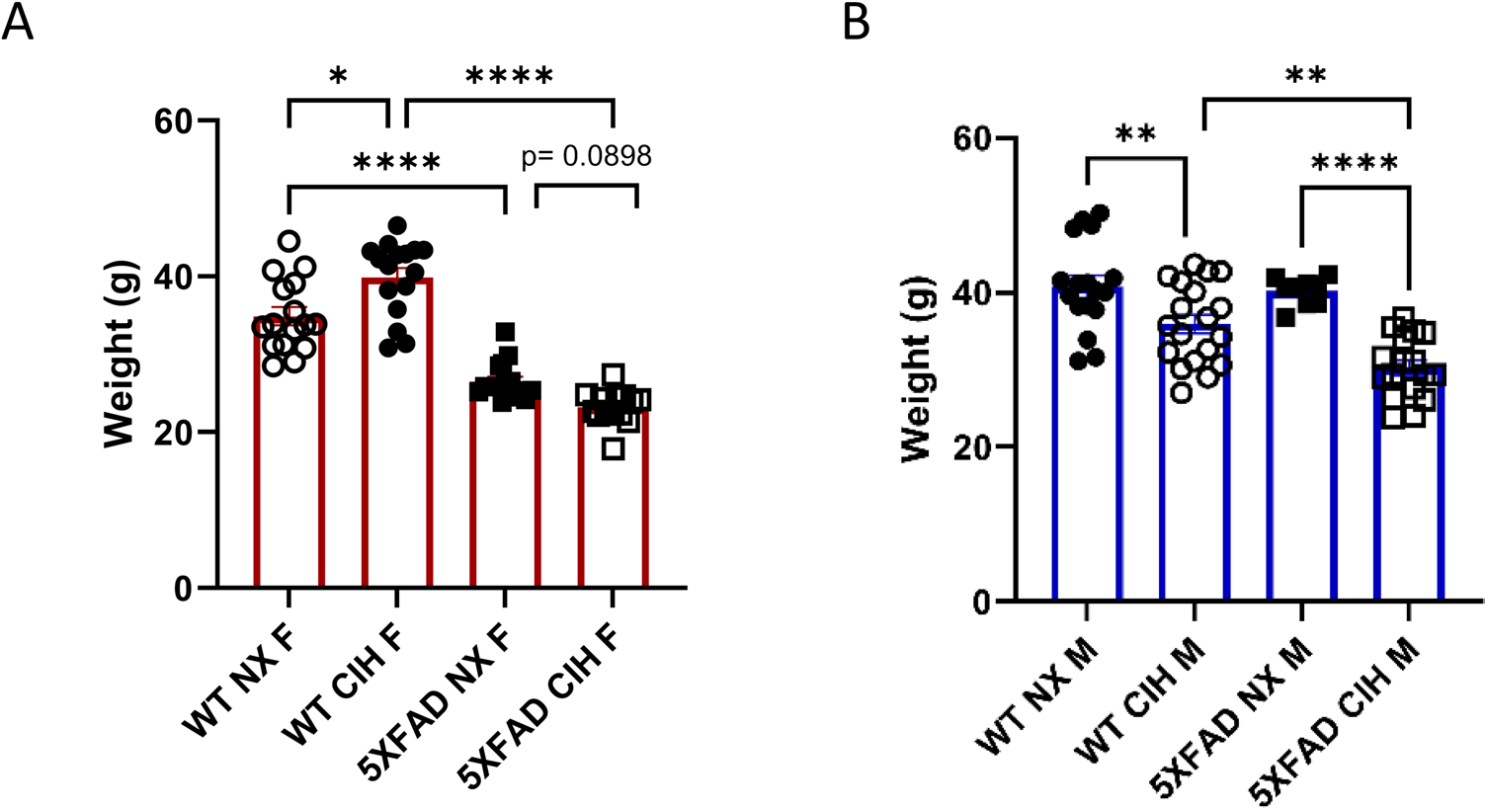
CIH exposure alters the weight of WT and 5XFAD mice. Weight (in grams) of female, 10-month-old (**A**) and male, 8-month-old (**B**) mice at harvest. Two-way ANOVA with Tukey multiple comparisons *p < 0.05, **p < 0.01, ***p < 0.001, ****p < 0.0001

Since metabolic dysfunction is associated with OSA and AD [1, 44–47], we recorded weight at the study endpoint. Females were sacrificed at 10 months of age and males at 8 months. In females, CIH exposure significantly increased the weight of WT mice, whereas female normoxia-exposed 5XFAD mice weighed significantly less than their WT counterparts, as expected (2-way ANOVA, *p* < 0.0001, Fig. 1A). Interestingly, CIH exposure tended to decrease the weight of 5XFAD females further, although this did not reach statistical significance (*p* = 0.0898). In contrast, in males, CIH exposure significantly decreased body weight in both WT and 5XFAD mice (2-way ANOVA, *p* < 0.0001; Fig. 1B), and there was no difference in body weight in NX-exposed WT and 5XFAD male mice at this age.

### Plaque Number and Size Are Unaffected by CIH in 8-Month-Old Males and 10-Month-Old Females

**Fig. 2:**
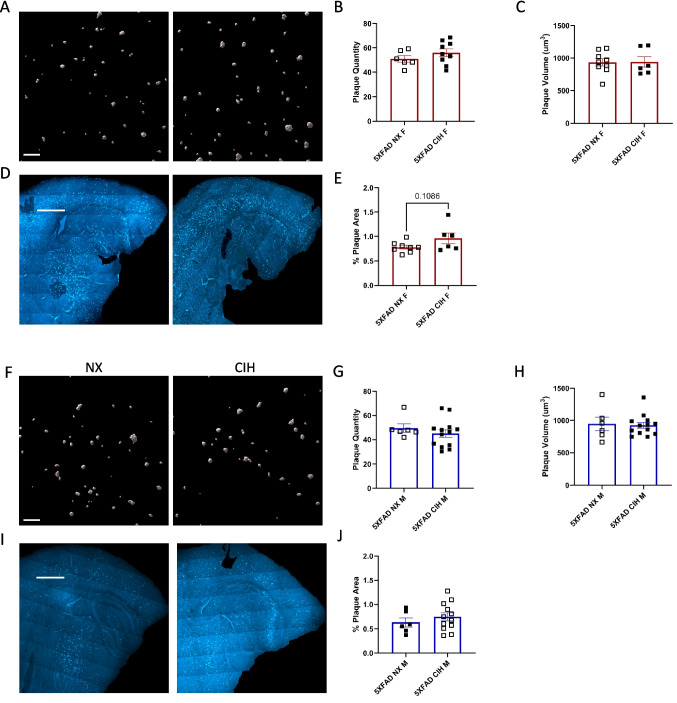
Plaque load and plaque morphology are unchanged with CIH exposure. Imaris surfaces generated from methoxy-X04 stained, confocal z-stack images of a high-power field in the cortex of NX or CIH treated 5XFAD female or male mice (**A**, **F**). Imaris quantification of the number of plaques per field and average plaque volume per image; each data point represents 6 images (**B**, **C**, **G**, **H**). Tiled images of normoxia or CIH treated 5XFAD female or male mice (**D**, **I**). ImageJ quantification of the percent area of the cortex that contains plaques (**E**, **J**; each point represents 3 sections per animal). There were no differences detected by unpaired *t*-test, *p* > 0.05. Scale bar is 70 µm (**A**, **F**) and 1000 µm (**D**, **I**)

Tissues were stained with methoxy-X04 to label β-amyloid plaques and imaged in 2 ways: (1) a Z-stack image of a high-powered field (HPF) in the cortex, and (2) a large, single-plane tiled image that encompassed the entirety of the cortex in that section. Images of the HPF were analyzed in Imaris (Bitplane) to quantify plaque number and characteristics. These analyses in 10-month-old female (Fig. 2A) and 8-month-old male (Fig. 2F) 5XFAD mice indicated no significant differences induced by CIH treatment either in the number of plaques present (Fig. 2B, G) or their size (Fig. 2 C, H) (unpaired *t*-test, *p* > 0.05). The large, tiled images (Fig. 2D, I) were quantified in ImageJ and are reported as a percent of the cortex ROI (Fig. 2E, J). Although not significant, there was a trend towards an increase in the total plaque area in the cortex of female 5XFAD mice that were exposed to CIH (unpaired *t*-test, *p* = 0.1086), but this difference was not observed in males (unpaired *t*-test, *p* > 0.05).

### CIH Does Not Affect Astrogliosis, but Trends Towards Decreasing Microgliosis with Amyloid Deposition in 5XFAD Mice

**Fig. 3:**
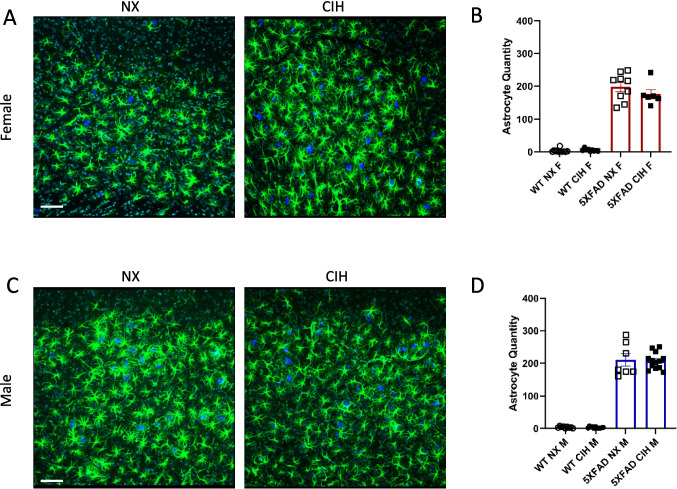
The astrocyte response to amyloid deposition is unchanged by CIH exposure. Sections were stained with anti-GFAP antibodies (astrocytes), TO-PRO-3 (nuclei), and methoxy-X04 (plaques) and then imaged by confocal microscopy (**A**, **C**). Images were analyzed in Imaris (Bitplane) by colocalization. Each point represents 3 images per animal. There was no significant effect of CIH exposure by two-way ANOVA with Tukey multiple comparisons; *p* > 0.05. Scale bar is 70 µm

Gliosis is an integral component of AD pathology, so we quantified the number of astrocytes and microglia per HPF in the cortex. Reactive (GFAP^+^) astrocytes were quantified in Imaris. Consistent with other reports [35, 48], there was an increase in reactive astrocytes in the cortex of both male and female 5XFAD mice when compared to WT (Fig. 3B, D; two-way ANOVA with Tukey multiple comparisons, *p* < 0.0001). However, CIH did not change the number of reactive astrocytes in either male or female 5XFAD mice compared to the NX-exposed counterparts (females, *p* = 0.6937; males, *p* = 0.6877).

**Fig. 4:**
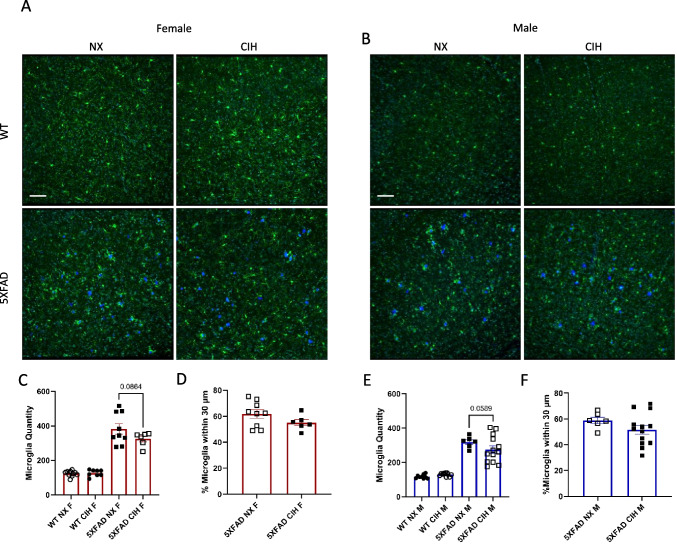
The microglial response is slightly depressed with CIH exposure. Representative images of cortical sections from NX and CIH WT and 5XFAD mice stained with anti-iba-1 (microglia), TO-PRO-3 (nuclei), and methoxy-X04 (plaques) and then imaged by confocal microscopy (**A** female, **B **male). Images were analyzed in Imaris (Bitplane) by colocalization (**C**, **E**). Microglia quantity and percentage of microglia 30 µm in female (**C**, **D**) and male (**E**, **F**) WT and 5XFAD mice exposed to NX or CIH. Each data point represents 3 images per animal. No significant effect of CIH exposure by two-way ANOVA with Tukey multiple comparisons, *p* > 0.05. Scale bar is 70 µm

The microglial response to amyloid deposition was quantified in the same manner. As expected, the 5XFAD genotype significantly increased microglia number in the cortex (two-way ANOVA, *p* < 0.001), and this tended to be decreased by CIH exposure in both males and females, although it did not reach statistical significance (two-way ANOVA, females, *p* = 0.0864; males, *p* = 0.0589; Fig. 4C, E). Further, since microglia cluster around plaques, an additional analysis of their relative localization was performed. Despite the trends in the overall number of microglia in each sex, there was no significant difference in the localization of microglia in relation to plaques between NX- and CIH-exposed female (unpaired *t*-test, *p* = 0.1632, Fig. 4D) or male (unpaired *t*-test*, p* = 0.2051, Fig. 4F) 5XFAD mice.

### CIH Drives Transcriptional Changes Related to Metabolism in Microglia

To investigate the transcriptional changes associated with CIH exposure in WT and 5XFAD mice, we performed bulk RNA sequencing analyses on immunomagnetically isolated microglia; differentially expressed genes (DEGs) were identified as those which had an FDR < 0.05 and Log_2_FC > 1. The total number of significant DEGs for each comparison is shown in Tables 1 and 2.

**Table 1:**
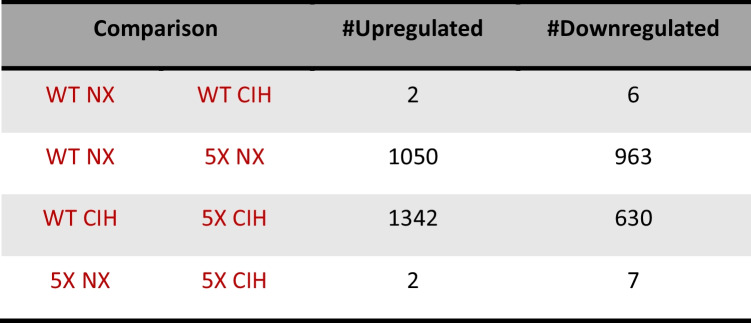
The number of significant DEGs in microglial RNA-seq data from female mice. Significant comparisons had an FDR < 0.05, LFC > 1

**Table 2:**
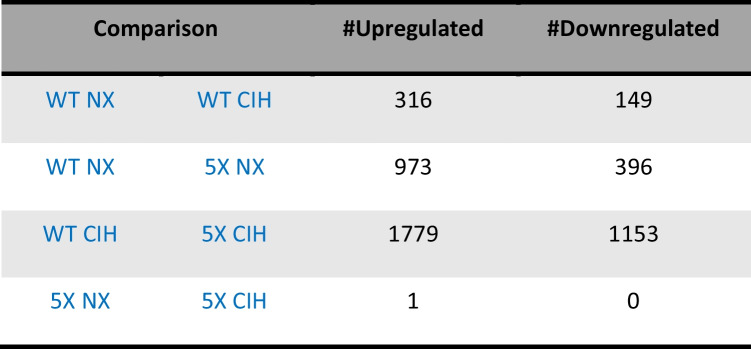
The number of significant DEGs in microglial RNA-seq data from male mice. Significant comparisons had an FDR < 0.05, LFC > 1

**Fig. 5:**
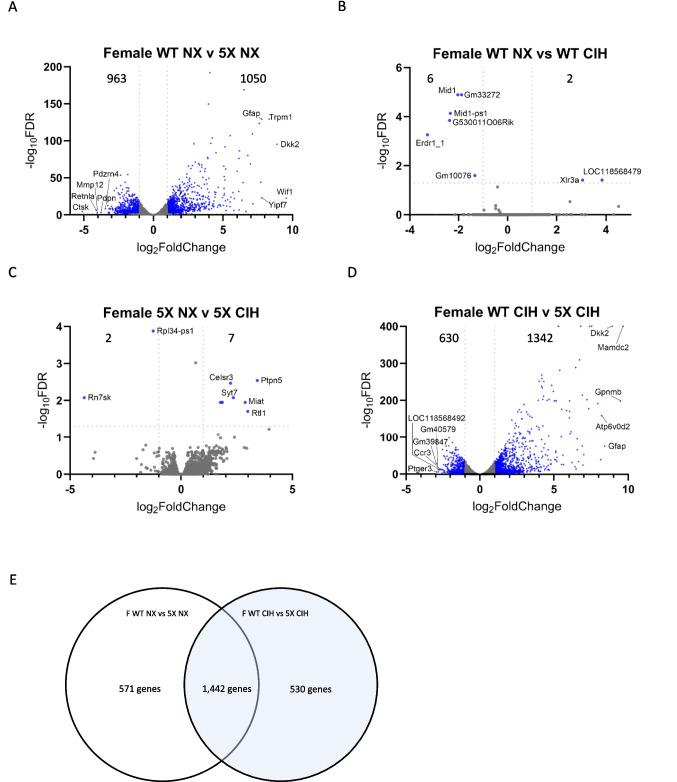

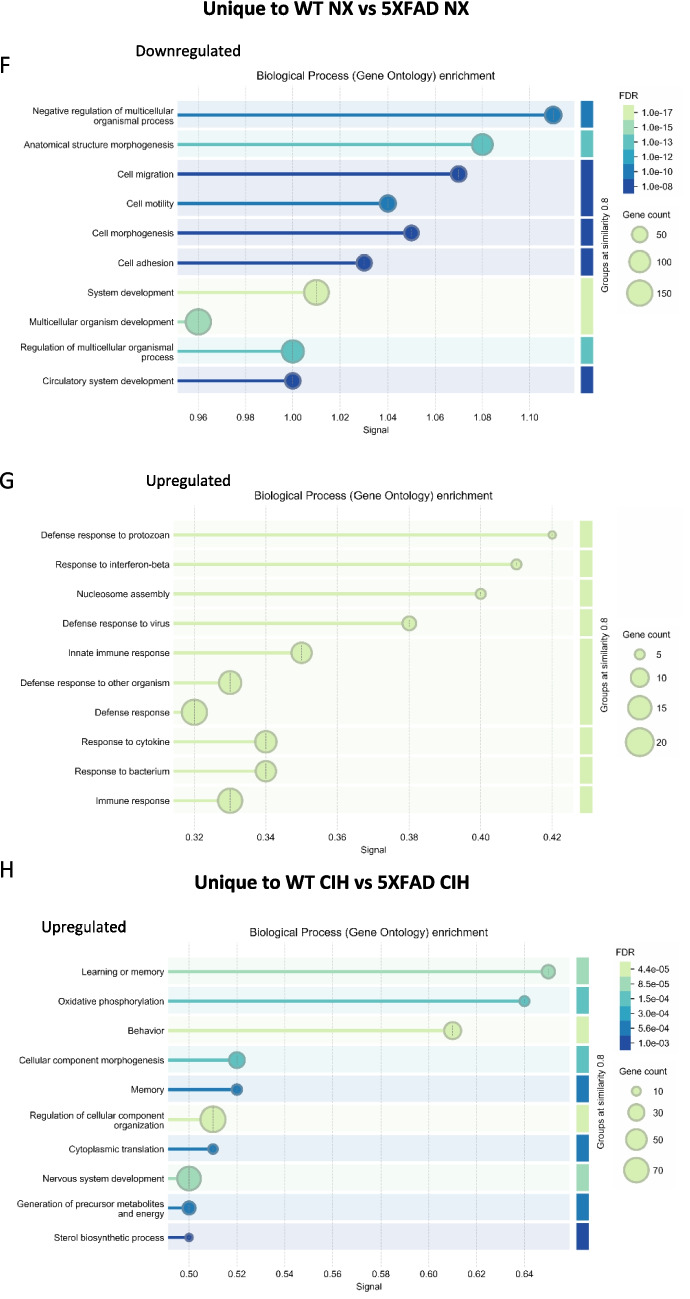
CIH exposure drives few changes in gene expression by RNA sequencing in WT females but induces a unique gene program in 5XFAD females. RNA was isolated from CD11b+ cells immunomagnetically isolated from the cortex of 10-month-old WT and 5XFAD female mice. Volcano plots with significant DEGs compared by genotype (**A**, **D**) and treatment (**B**, **C**) for all genes with FDR < 0.05 and LFC > 1. Venn diagram showing the number of overlapping and unique genes between the female WT and 5XFAD comparisons in NX and CIH treatment (**E**). Biological processes gene ontology (GO) analyses of those genes specific to the female WT NX vs 5XFAD NX comparison that are downregulated (**F**) and upregulated (**G**). Biological processes GO analyses of those genes specific to the female WT CIH vs 5XFAD CIH comparison that are upregulated (**H**)

We first analyzed the effect of genotype in microglia from NX-exposed females, by comparing WT and 5XFAD microglia (WT NX vs 5XFAD NX; Fig. 5A). There were 2013 significant DEGs; 1050 were upregulated and 963 were downregulated (see supplemental file 1 for full gene list). These DEGs included genes that characterize disease-associated microglia (DAM) [49], as well as pathways related to TREM2-APOE [50] and aging [51]. Next, we compared the effect of CIH on WT female microglia (WT NX vs WT CIH), and 8 genes were differentially expressed: *LOC118568479* was the most upregulated gene (LFC = 3.84) and *Erdr1_1* was the most downregulated (LFC = −3.28, volcano plot, Fig. 5B). Comparing the effect of CIH on microglia from 5XFAD females (5XFAD NX vs 5XFAD CIH), there were a total of 9 DEGs; 7 were upregulated. The most upregulated was *Ptpn5* (LFC = 3.42), and of the 2 downregulated genes, the greatest change was in *Rn7sk* (LFC = −4.35) (volcano plot, Fig. 5C). We next assessed whether CIH had differential effects on microglia from WT females compared to 5XFAD females (WT CIH vs 5XFAD CIH; Fig. 5D). There were over 1000 genes up- and downregulated (see supplemental file 1 for full gene list), which, like the normoxia comparison, were due primarily to the effect of the 5XFAD genotype and consistent with those reported by us and others [48, 49]. To further examine the effect of CIH with respect to the microglial response to plaque pathology in female 5XFAD, we cross-referenced the DEGs between the WT NX vs 5XFAD NX and WT CIH vs 5XFAD CIH comparisons (see Supplemental file 3). 1442 genes were shared between the comparisons; 571 genes were specific to the WT NX vs 5XFAD NX comparison, and 530 genes were unique to the WT CIH vs 5XFAD CIH analysis (Fig. 5E). We performed gene ontology (GO) analyses on the up- and downregulated genes that were unique to each respective comparison. The DEGs that were uniquely downregulated in the WT NX vs 5XFAD NX comparison were most enriched for system development, multicellular organism development, and anatomical structure development, as well as circulatory system development and cell motility, migration, morphogenesis and adhesion (Fig. 5F). The DEGs that were uniquely upregulated in the WT NX vs 5XFAD NX comparison included enrichment for the defense response, the innate immune response, and the response to interferon-β (Fig. 5G). When we examined the DEGs that were unique to the WT CIH vs 5XFAD CIH comparison, no GO terms were identified for the downregulated genes. However, those that were uniquely upregulated in the WT CIH vs 5XFAD CIH comparison were significantly enriched for the GO terms associated with behavior and memory, regulation of cellular components, oxidative phosphorylation and precursor metabolite generation (Fig. 5H). In all, the comparisons between the effect of genotype and each respective treatment highlight that CIH can influence processes associated with metabolism, learning and memory.

**Fig. 6:**
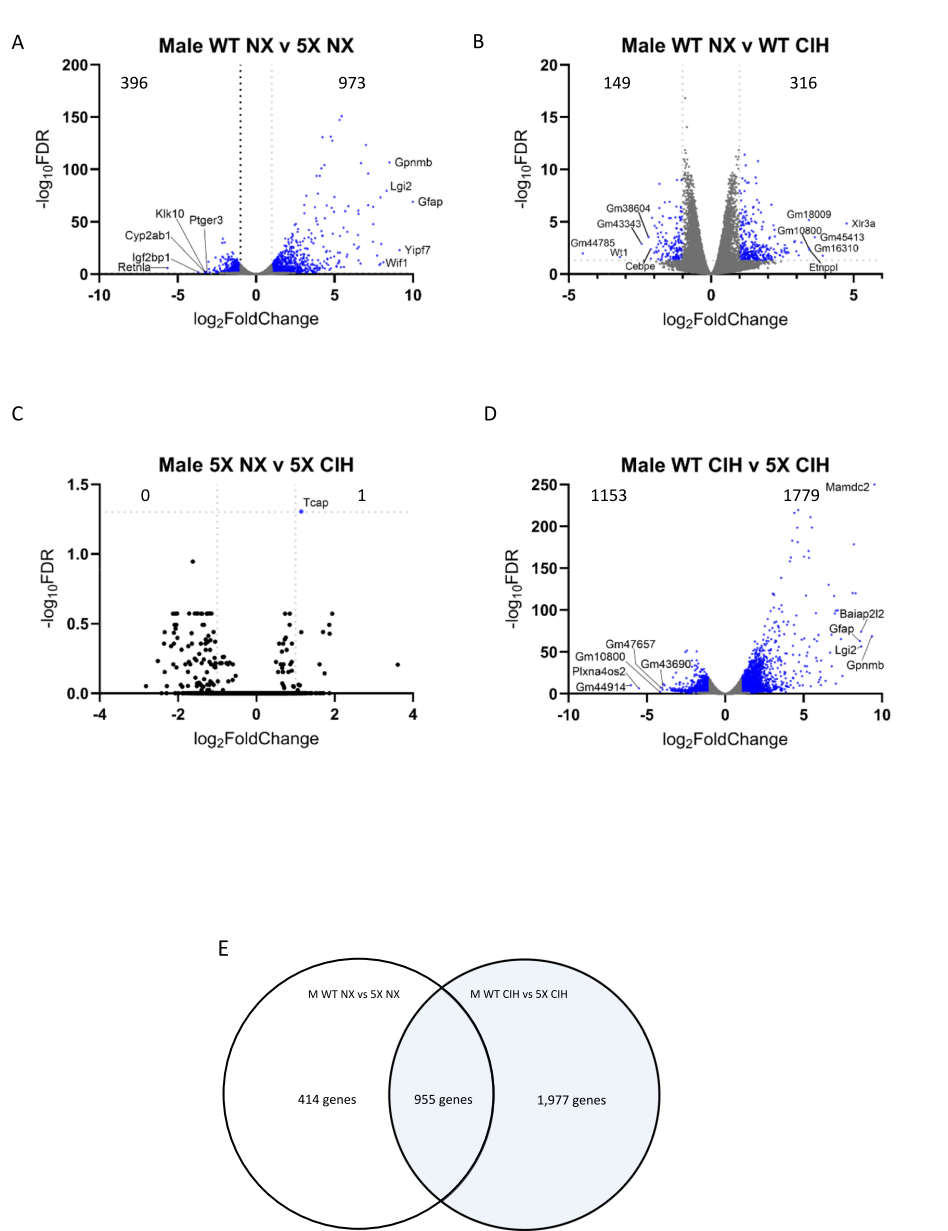

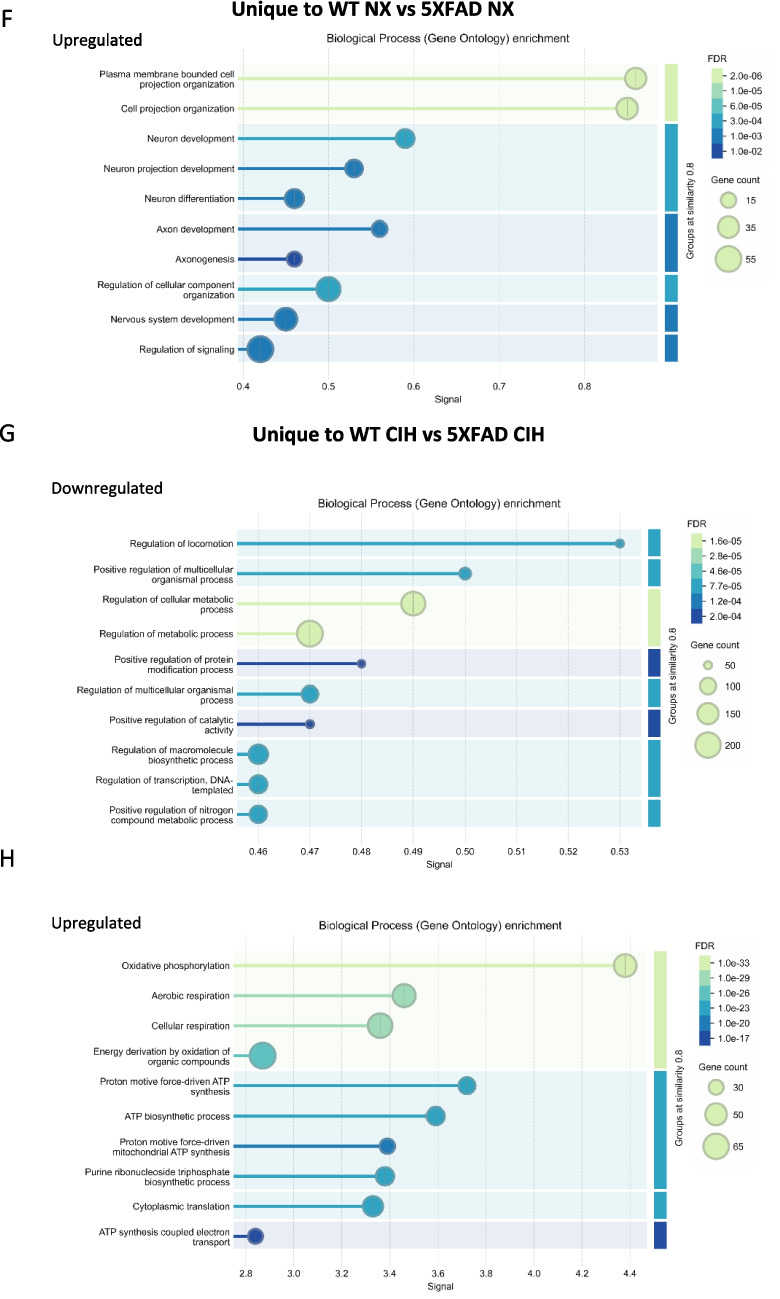
CIH exposure in males drives significant differential expression by RNA sequencing. RNA was isolated from CD11b+ cells immunomagnetically isolated from the cortex of 8-month-old WT and 5XFAD male mice. Volcano plots with significant DEGs compared by genotype (**A**, **D**) and treatment (**B**, **C**) for all genes FDR < 0.05, LFC > 1. Venn diagram showing the number of overlapping and unique genes between the male WT and 5XFAD comparisons in NX and CIH treatment (**E**). Biological processes gene ontology (GO) analyses of those genes specific to the male WT NX vs 5XFAD NX comparison that are downregulated (**F**) and upregulated (**G**). Biological processes GO analyses of those genes specific to the male WT CIH vs 5XFAD CIH comparison that are upregulated (**H**)

We used the same analysis pipeline to examine the data from male microglia. The 5XFAD genotype had a significant effect on NX-exposed animals, with 973 genes being upregulated and 396 downregulated. These changes included the key genes associated with the 5XFAD genotype (Fig. 6A; see supplemental file 2 for the full gene list). We then analyzed the effect of CIH on microglia from WT animals. CIH had a more significant effect on WT male microglia than it did on WT female microglia. In WT males, a total of 465 genes were differentially expressed; 149 were downregulated and 316 were upregulated (Fig. 6B). Those that were significantly decreased included the type-I interferon associated neuroinflammatory genes *Cxcl10* (Log_2_FC = −1.20), *Ccl5* (Log_2_FC = −1.18), *Ifitm2* (Log_2_FC = −1.17), *Ifitm3* (Log_2_FC = −1.25), and *Ifitm6* (Log_2_FC = −1.60). The *Xlr3a* gene was the most upregulated (Log_2_FC = 4.76). Notably, several genes encoding solute and amino acid transporters were significantly upregulated (see supplemental file 2 for the full gene list). We then compared the effect of the 5XFAD genotype on microglia from CIH-exposed males. In the comparison of CIH-exposed WT and 5XFAD mice, over 2900 genes were differentially expressed; 1779 were upregulated and 1153 were downregulated (Fig. 6C), compared to less than 1400 genes that were differentially expressed due to the 5XFAD genotype in NX-exposed males (Fig. 6A). CIH alone had little to no effect on the male 5XFAD microglial transcriptome compared to NX; only 1 gene (*tcap* (Log2FC = 1.15)) was differentially upregulated (Fig. 6D). While the comparison of CIH-treated WT and 5XFAD mice still included the hallmark changes associated with the 5XFAD genotype, key DAM genes that were upregulated in 5XFAD NX mice were less upregulated upon CIH exposure, suggesting that CIH may dampen the DAM gene profile in the microglia of male 5XFAD mice.

To further examine alterations to the DAM profile in males, we investigated the effect of the 5XFAD genotype in microglia from NX-exposed male WT and 5XFAD mice (Fig. 6E, left) and the effect of CIH on male WT and 5XFAD mice by cross-referencing all of the DEGs (up- and downregulated) between these comparisons (Fig. 6E). Surprisingly, 955 genes were shared between them, though there were 1965 genes unique to the WT CIH vs 5XFAD CIH comparison and 414 genes that were unique to the WT NX vs 5XFAD NX comparison (see Supplemental file 3). We analyzed these differences using a gene ontology (GO) enrichment analysis, and a summary of the top 10 significantly enriched pathways is presented in Figs. 6F-–H. None of the genes that were unique to the WT vs 5XFAD NX comparison was downregulated. However, the upregulated genes fell into categories associated with pathways involved in cell projection and neuron and axon development (Fig. 6F). The genes that were uniquely decreased in microglia from the WT CIH vs 5XFAD CIH comparison were enriched for gene categories involved in locomotion, cellular metabolic processes, protein modification, catalytic activity, macromolecule biosynthesis, transcription and nitrogen compound metabolism (Fig. 6G). Notably, the upregulated genes unique to the WT CIH vs 5XFAD CIH comparison were enriched for GO pathways associated with oxidative phosphorylation, aerobic and cellular respiration and ATP synthesis (Fig. 6G). Together, comparisons between the effect of genotype and treatment highlight the idea that CIH treatment alters oxidative phosphorylation and cell respiration, among others, in 5XFAD microglia. Interestingly, the DEGs in 5XFAD microglia from the NX comparison, but not the CIH comparison, suggest that microtubule structure and microglial morphology may be more impacted by the 5XFAD genotype, although the significance of this requires additional investigation.

## Discussion

There is a strong clinical correlation between AD and OSA. But despite a high prevalence of both disorders, their relationship remains poorly understood. Insights into this interaction could be leveraged as an early intervention strategy to mitigate or lessen future cognitive decline. Accordingly, the goal of our experiments here was to study the effects of the CIH associated with sleep disordered breathing in an animal model of early amyloid pathology. We initiated CIH exposure at 4 months of age, an early stage of pathology in the 5XFAD mouse model, and continued it daily until sacrifice. As we expected, we found that CIH exposure reduced body weight in 5XFAD male and female mice. In contrast to our initial hypothesis, however, the combination of amyloid pathology and CIH did not significantly alter amyloid plaque deposition (Fig. 2), nor the number of astrocytes (Fig. 3) or microglia (Fig. 4) in the cortex. CIH also did not alter the way microglia clustered around amyloid plaques, in 5XFAD of either sex. But as expected, there were significant transcriptomic changes induced by CIH exposure in microglia from WT animals, although this is the first report of these transcriptomic alterations in murine microglia. CIH exposure also significantly altered the microglia transcriptome associated with amyloid pathology compared to CIH exposure in WT microglia (Figs. 5 and 6). Few DEGs were induced by CIH in 5XFAD microglia (compared to 5XFAD NX-exposed microglia) from either female (Fig. 5) or male (Fig. 6) mice, likely because the effect of the 5XFAD genotype is a stronger driver of transcriptomic changes in microglia than CIH.

We chose to model the intermittent hypoxia aspect of SDB in the present studies. Although intrathoracic pressure swings, sleep fragmentation, intermittent hypercapnia, and augmented sympathetic drive (among others) also characterize SDB [52, 53], most SDB morbidities can be modeled in rodents with CIH exposure alone [54, 55]. The use of CIH in animal models permits mechanistic investigations of individual SDB features that are not possible in humans where multifactorial traits cannot be readily disentangled. The duration, interval and severity of the intermittent hypoxia are critical determinants of its potential to exert neuroprotective or pathologic effects [56–58]. However, a limitation of this CIH model is that it is unclear whether the mice also experienced sleep fragmentation [29, 59], and if so, for how long, as this is also thought to contribute to cognitive decline in the aging population [60, 61] and may interact with amyloid pathology [62]. Further, recent work suggests that cognitive changes associated with CIH in mouse models may be mediated by activation of the NLRP3 inflammasome [63], a major mediator of inflammation that accompanies AD and aging [64]. In fact, NLRP3-mediated neuroinflammation is thought to reduce microglial phagocytosis of Aβ [65], providing another potential link between AD and OSA co-morbidity.

Previous data suggests a significant interaction between OSA and AD in other AD-like models of amyloidosis, exposed to a much shorter duration of CIH (a few days or weeks vs 4 or 6 months in our studies). For example, in 10–11-month-old APP/PS1 mice, exposure to CIH for 4 weeks induced astrogliosis in the cortex [34]. Further, recent work using the APP NL-G-F knock-in mouse identified changes in Aβ, hyperphosphorylated tau, and β-Secretase 1 (BACE-1) in male mice 2 weeks following a 4-day CIH protocol [66]. Multiple groups have identified spatial learning and memory deficits induced by CIH based on reductions in behavioral performance, postsynaptic density, and long-term potentiation in the hippocampus in two separate AD model paradigms: APP/PS1 mice after 2 weeks of CIH treatment and a tau mouse model after 8 weeks of CIH exposure [67, 68]. When compared to our findings, these data support the idea that changes in the neuroinflammatory response may be dependent on the CIH exposure time, as we did not identify changes in pathology or gliosis by microscopy in the much longer, multi-month CIH timepoints we assessed here. Mice are incredibly adaptable to hypoxia, reducing their metabolic rates to adapt to the low oxygen environment. Because of this, we chose to expose them to 6% inspired O_2_ for 12 h each day. In addition, the CNS is highly plastic and can adapt to compensate for pathologic insults, especially after several months of CIH exposure, as used here. We have previously shown that this CIH paradigm induces neuronal death in the mouse hippocampus within 2 weeks of CIH exposure, and by 1 month, neuronal loss is no longer detectable [33]. This has been suggested to be due to compensatory neurogenesis induced by CIH [29, 69]. Future work could include longitudinal behavioral testing to distinguish the temporal dynamics of CIH exposure and CNS adaptation mechanisms in mice.

Previous work by us and others [38, 70] suggests the effects of CIH are sex-dependent. In the present report, females demonstrated few microglial transcriptomic changes when the effects of CIH treatment were compared to NX in 5XFAD animals. However, analysis of the comparisons made between WT and 5XFAD mice in the CIH condition identified an enrichment of GO terms associated with learning and memory as well as oxidative phosphorylation in the upregulated DEGs. These changes suggest there may be an effect of CIH exposure on metabolic processes, and may hint at a mechanism for the behavioral changes often associated with AD [48]. The differential expression of pro-inflammatory genes in isolated microglia from WT males following CIH treatment aligns with what our group and others have shown in both in vitro and in vivo models ([32], reviewed in [33]). The comparison of NX vs CIH treatment of 5XFAD male mice only resulted in the identification of a single DEG (*tcap*) which, given current literature, does not clarify how CIH and the 5XFAD genotype interact. More DEGs may be identified if there is a critical window for interaction between CIH treatment duration and amyloid pathology progression. To further investigate this interaction in our existing dataset we analyzed the comparisons made between WT and 5XFAD mice in the NX and CIH conditions. Notably, CIH appeared to dampen the DAM profile established in 5XFAD microglia. The identification of GO pathways associated with oxidative phosphorylation and ATP synthesis in 5XFAD CIH mice versus the WT CIH comparisons, but not within the NX genotype comparisons, may suggest that CIH influences the metabolic response of microglia.

As AD and SDB pathologies become more prevalent, the processes underlying their clinical association become of increasing importance. We sought to model SDB during the early accumulation phase of amyloid pathology. While there were no gross pathologic changes detected in the cortex of male or female 5XFAD mice, the effects on the microglia transcriptome, particularly in metabolic pathways, were mechanistically informative. Given the sex differences in OSA (males disproportionately affected) [70] and AD (females disproportionately affected) [71] and that there was an interaction between CIH and 5XFAD on the microglial transcriptome, it is intriguing and will require future analyses of potential hormonal contributions as the mice in the present study were gonadally intact. In sum, our results highlight the need for and importance of additional studies to examine the effect of longer-term CIH exposure on later stages of AD pathology using tau mouse models, the role of gonadal hormones, and investigating other aspects of SDB beyond CIH, such as sleep fragmentation.

## Supplementary Information

Below is the link to the electronic supplementary material.

ESM 1 (XLSX 342 KB)

ESM 2 (XLSX 437 KB)

ESM 3 (XLSX 1.01 MB)

## Data Availability

Sequencing data are available on GEO (accession #GSE272491). Please contact authors to request any additional data.
